# Understanding online health information seeking behavior of older adults: A social cognitive perspective

**DOI:** 10.3389/fpubh.2023.1147789

**Published:** 2023-03-03

**Authors:** Xiumei Ma, Yunxing Liu, Pengfei Zhang, Rongtao Qi, Fanbo Meng

**Affiliations:** ^1^Faculty of Business, Hong Kong Polytechnic University, Kowloon, Hong Kong SAR, China; ^2^Industrial Design Department, Eindhoven University of Technology, Eindhoven, Netherlands; ^3^School of Political Science and Public Administration, Soochow University, Jiangsu, China; ^4^School of Business, Jiangnan University, Wuxi, Jiangsu, China

**Keywords:** online health information seeking, older adults, social cognitive theory, health awareness, perceived benefit

## Abstract

**Introduction:**

Online health information seeking has been verified to play a crucial role in improving public health and has received close scholarly attention. However, the seeking behavior of older adults, especially the underlying mechanism through which they are motivated to seek health information online, remains unclear. This study addresses the issue by proposing a theoretical model leveraging social cognitive theory.

**Methods:**

IT self-efficacy and IT innovativeness were identified as personal factors and professional support and social support were identified as environmental factors. We conducted a survey that included 347 older people in China and examined the research hypotheses with a structural equation model.

**Results:**

IT self-efficacy and IT innovativeness facilitate older adults to seek health information online by increasing their perceived benefit of using the internet. Additionally, professional support and social support enhanced older adults' online seeking behavior by promoting their health awareness. We also found that perceived benefit displayed a stronger impact than health awareness on older adults' behavior related to searching for health information online.

**Conclusion:**

This study reveals that IT self-efficacy, IT innovativeness, professional support, and social support will promote older adults to seek health information online by enhancing their health awareness and perceived benefit. The findings of this study provide significant theoretical and practical implications.

## 1. Introduction

The rapid development of information technology has made the internet the most popular source of health information based on convenient access and quick response, and the number of people making use of the internet to search for health information has continued to grow. National surveys conducted in America, German, China and other countries indicated that a large proportion of internet users frequently searched for health information, totaling more than 50% of the respondents in those countries (1). Especially in the context of the COVID-19 epidemic, the internet has become the primary means as well as the best way to obtain health information.

Health information seeking behavior has been the subject of scholarly attention since the 1960's. Recently, the advent of the information technology age and internet growth have focused the spotlight on online information seeking concerning health-related topics. The term online health information seeking refers to individuals using the internet to search for find information about their health, risks, illnesses, and health-protective behaviors. Ease of access, immediacy, and diversity of information sources are all factors that have contributed to individuals choosing online information as their preferred source for health information (2). In fact, empirical evidence demonstrates significant impacts of internet-based health information on individuals' physical and psychological health (3, 4).

In particular, scholars are paying increasing attention to older adults' behaviors related to seeking health information online (5–7). According to the World Health Organization, the population of adults over age 60 will reach 2.1 billion by 2050^1^ As a result of the aging of the world's population overall, the tremendous growth of healthcare and health information needs has become a significant issue. Although seeking health information online is beneficial in addressing or solving health problems, its popularization among older adults faces many challenges due to these individuals' generally lower cognitive and technical abilities. For example, many older adults suffer a lack of technical skills and internet search skills that impedes them from using the internet to access information (8). In addition, some research has indicated that older adults tend to rely on their cognitive ability and existing medical knowledge when seeking health information; however, their cognitive abilities have been shown to typically decline with age (9). Therefore, identifying factors that can support older adults to search for health information online has major implications in both practice and research.

Although prior studies have made a great effort to identify the determinants of online health information seeking behavior (10–12), investigations into better ways to support and encourage older adults' search behavior remain scarce, and the underlying mechanisms are yet unclear. For example, some researchers identified such instrumental factors as information quality, trustworthiness, and utility of information as the dominant predictors of online health information seeking (1). However, in the case of older people, obstacles to searching for information online include a having negative attitude about the internet, entertaining poor health beliefs, and suffering IT deficiencies and low support from others (5). Thus, instead of concentrating on factors related to the health information itself, studies should pay more attention to the individual and environmental factors that directly affect older adults, such as individual cognition, IT resources, and external support. Furthermore, attaining deeper understanding of how to support older adults in searching for health information online requires exploring the mechanism through which these factors influence their health information seeking behavior.

This study addressed the above issues by drawing upon social cognitive theory to develop a theoretical research model. Two reasons support the choice of this theory for the current study. First, social cognitive theory has been widely used to explain and predict individuals' behavior and decision-making, especially in the contexts of health behaviors and information behaviors (13, 14). Second, social cognitive theory illustrates how personal factors and environmental factors simultaneously influence individual behavior (15), which fits well with the current study's research objective. In this study, we conceptualized IT efficacy and IT innovativeness as observable personal factors and conceptualized social support and professional support as the environmental factors of interests. Next, we examined how both types of factors influenced the online search behavior of older adults seeking health information through increasing their perceived benefit of using internet and their health awareness.

This study makes several contributions to the field. For example, the findings provide a new understanding of how older adults seek for health information online from the perspective of social cognitive theory. In particular, this work is one of the first to empirically investigate how the targeted information seeking behavior is influenced by older adults' individual IT capacity and environmental support. In addition, this study clarifies the influencing mechanism through introducing the specific cognitions of perceived benefit and health awareness. This outcome fills the gap left by previous studies that focused primarily on exploring influencing factors while overlooking the underlying mechanism. Lastly, this study verifies specific personal factors and environmental factors affecting older adults based on their characteristics in the context of searching for health information online. This aspect of the current study thus complements the existing research while also providing practical suggestions on how to improve older adults' efforts on looking for health information online.

## 2. Literature review and theoretical background

### 2.1. Online health information seeking of older adults

The development of information technology has caused the internet to become the main source for health information seeking (16); as a result, many studies have focused on online health information seeking behavior. Scholars have used multiple perspectives to investigate this topic and verified its crucial role in healthcare. For example, Zhao and Zhang (17) found that health-seeking on social media could fill the demand for health information while also providing social and emotional support *via* peer-to-peer interaction.

Notably, the growth in the number of older adults and their high prevalence of health problems has attracted scholars' close attention to older adults' information seeking behavior. However, even though the positive results reported for online health information seeking, studies found that using the Internet to obtain health information is comparatively low among older adults (18). For example, research found that older adults have less trust in the Internet source and present negative attitudes toward health information from Internet (19). Moreover, older age and reduced cognitive abilities hinder older adults' access to the Internet and online health information (20). In fact, a current study illustrated that older adults relied on medical personnel, family and friends, and health brochures rather than the Internet as main sources of health information (7). Existing findings indicate that it is necessary to help older adults better utilize the Internet to search for health information.

Recent studies have made great efforts to explore the motivators facilitating online health information seeking behavior of older adults. For example, Oh and Lim (21) found that communication with medical professionals significantly accelerated use of the Internet by older people to search for health information. Weber et al. (7) found that older adults' seeking behavior is related to their lifestyle, where the Average Family Person and the Sociable Adventurer use the internet more often for health information. In addition, research has found that the health condition, especially a recent diagnosis of cancer, positively facilitated older people to seek health information on the Internet (22). An empirical study by Zhu et al. (23) revealed that social support, and self-efficacy were necessary predictors of health information seeking for older adults with coronary heart disease.

However, although the current literature offers insight into factors influencing online health information seeking of older adults, few of the prior studies have yet clarified the influence mechanism (24). Improving the online seeking behavior of older people is more difficult than that of younger people because of cognitive limitations, low electronic health literacy, and negative attitudes toward technology (6). Thus, in addition to exploring the influencing factors, understanding the mechanism of influencing factors can fundamentally provide evidence for effectively promoting the online health information seeking behavior of older adults. According to previous findings, older adults' IT-related capabilities and support from their external environment are key factors affecting their behavior to search for health information online (25, 26). Consequently, this study aimed to deeply revel how personal IT-related factors and environmental factors might influence older adults' behavior in terms of searching for health information online.

### 2.2. Social cognitive theory

Social cognitive theory, a classical theory that finds its basis in social learning theory, has been widely used to explain individual actions (27). This theory can be referred to as ternary reciprocal determinism; in other words, individual behaviors are determined by the interaction of three factors: person, environment, and behavior (28). In addition, these three factors can influence each other, and any two factors can influence the third factor (28). Since Bandura originally proposed the social cognitive theory, it has received ongoing examination with a focus on various individual behaviors.

The growing prominence of health issues has led to the argument that social cognitive theory should be used to achieve a healthy society (29, 30). In fact, social cognitive theory has been one of the most influential theories on health behavior (31). In particular, the key construct of social cognitive theory, self-efficacy, has been incorporated into most health behavior theories (32). Social cognitive theory addresses the environmental determinants of health as well as personal determinants and has been widely applied in the study of older adults' health behaviors and health management. For instance, based on the social cognitive theory, Borhaninejad, Iranpour (33) found that self-efficacy, social support, outcome expectations, and outcome expectancy significantly predicted diabetes self-care behaviors among the older people. In a similar vein, Zhang et al. (34) used social cognitive theory to investigate the impact of information communication technology usage on older adults' loneliness; in their findings, the authors identified the crucial role of self-efficacy and health awareness. Social cognitive theory was also successfully applied in predicting respiratory infection prevention among older adults (35).

Existing research suggests that social cognitive theory is suitable for the study of health behavior of older adults. Although many studies have incorporated social cognitive theory, few scholars have used social cognitive theory to explore older adults' online health information seeking. In contrast, this study focused on the determinants of older adults' behaviors related to searching for health information online, giving additional attention to individual differences and environmental uncertainties. Consequently, this paper addresses the identified research gap by proposing a research model based on social cognitive theory to understand how older adults approach seeking for health information online.

## 3. Research model and hypotheses development

**Figure 1:**
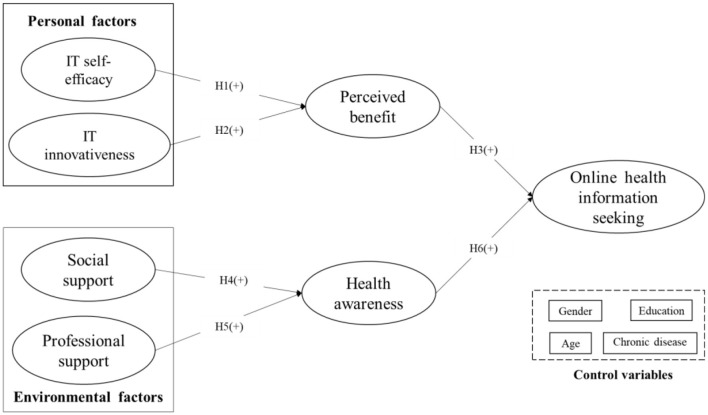
Theoretical research model.

Based on the social cognitive theory, this research took personal factors (self-efficacy and IT innovativeness) and contextual factors (social support and professional support) as antecedent variables in developing a research model to verify how personal and environmental factors influence older adults' online information seeking *via* perceived benefit and health awareness. In addition, gender, age, education level, and the existence of chronic disease were included as control variables. An illustration of the proposed research model appears in Figure 1.

### 3.1. Personal factors

Self-efficacy, widely recognized as a critical factor that affects individual behavior, generally refers to the determination and belief that individuals can complete an action under specific circumstances and can also refer to an individual's assessment of self-ability (36, 37). The current study refers to IT self-efficacy as older adults' judgment of their ability to use information technology to locate health-related information. Older people with high IT self-efficacy are likely to experience smooth, enjoyable internet interaction and will probably obtain positive outcomes (38). Some previous studies have confirmed that self-efficacy can positively affect users' perceived value (39, 40). In the context of obtaining health information from an online source, older adults who have mastered the necessary skills to use information technology tend to access valuable health information easily and are more likely to perceive that using the Internet to seek health information is beneficial. Thus, our first hypothesis is based on the assumption that IT self-efficacy will positively affect older adults' perceived benefits.

**H1:** IT self-efficacy is positively related to perceived benefit.

As another consideration, innovativeness generally refers to the degree to which a person prefers to use new technologies, products, or services (41). IT innovativeness can be defined as the willingness of an individual to try out any new information technology (42). For the purposes of this study, IT innovativeness as a personal trait represents older adults' tendency to focus on and accept new information technologies or new IT functions. Prior findings suggest that individuals with higher innovativeness are able to cope with a higher level of uncertainty (43). Thus, it is reasonable to expect that a high level of IT innovativeness leads to positive experiences and outcomes from using IT. Previous research has also noted that personal innovativeness has a strong positive effect on perceived ease of use and perceived benefit (41, 44, 45). Similarly, older people who are more willing to accept and use new information technologies will probably perceive greater benefit during the health information seeking process. These observations form the basis for our second hypothesis, as follows:

**H2:** IT innovativeness is positively related to perceived benefit.

Perceived benefit refers to consumers' confidence that they can improve their circumstances by using certain products or services (46). In this research, perceived benefit specifically refers to older adults' perception of positive consequences by using information technologies or the Internet to seek health information. Perceived benefit is usually regarded as relative advantages, which have the capacity to meet individuals' needs or wants and further positively influence their behavior (47). Thus, it is reasonable to predict that when individuals perceive beneficial outcomes from certain behaviors, they are more likely to continue the behavior. Several previous studies have provided empirical evidence of perceived benefit significantly facilitating user behavior (48, 49). In this study, when older adults perceive that using the Internet to seek health information can meet their needs conveniently and in a timely way, their seeking behavior is likely to be encouraged. Accordingly, we proposed the following in our third hypothesis:

**H3:** Perceived benefit is positively related to online health information seeking.

### 3.2. Environmental factors

Social support has become an essential predictor of online health information seeking (23, 50). Social support refers to people's access to various resources provided by others through interpersonal communication, including support concerning information, assistance, and comfort (51). In this study, we specifically define social support as resources and support from family, friends, and other non-professional social peers. Scholars have widely verified that social support exerts a significant impact on individuals' health attitudes and decisions. For example, individuals will be more aware of making healthier lifestyle decisions when they receive social support through interpersonal communication (52). In addition, Choi (53) discovered that social support, such as the care of family members, will encourage individuals to actively participate in their own health maintenance. Health awareness is the consciousness to maintain one's health; thus, it is reasonable that social support is positively related to health awareness, leading to the formulation of the study's fourth hypothesis, as follows.

**H4:** Social support is positively related to health awareness.

Professional support means that individuals obtain help from professionals who have received professional training or education in medicine and healthcare (54). In this study, it distinguishes social support and refers to support and help from medical professionals. Since health professionals are required to master qualified and effective health knowledge, they play a crucial role in helping individuals establish positive health views, change health behaviors, and attain improved health outcomes (55, 56). When individuals receive professional support from doctors, their health problems are likely to be effectively resolved. Especially in the case of older adults, communication with health professionals enables them to access reliable medical knowledge and update their understanding of health issues, which will likely enhance their health awareness. Based on these ideas, we proposed the following:

**H5:** Professional support is positively related to health awareness.

Health awareness is generally used to measure the readiness of individuals to take health actions (57). People with higher health awareness tend to be more active and pay more attention to health information than their peers (58, 59). Previous studies have identified health awareness as a driver leading to healthy lifestyle change and able to affect health-related behaviors (60, 61). In addition, some scholars have suggested that the higher the level of an individual's health awareness, the more concerned the individual will be about his or her health, motivating the person to further engage in health-promoting behaviors (62). For the purposes of this study, online health information seeking is regarded as a kind of health behavior. Thus, it is reasonable to argue that people with higher health awareness will more actively seek health information. This argument supports the following proposal:

**H6:** Health awareness is positively related to online health information seeking.

## 4. Methodology

### 4.1. Data collection

To test the research model, we collected data *via* a survey that targeted to older adults who had experience using information technology to seek health information. Because the survey was conducted in mainland China, we employed the backward translation method to translate the questionnaire into the Chinese language. Before data collection began, a pilot test was conducted to ensure that the measurement would be clear and understandable to participants. According to the pilot test results, along with comments and feedback from the interviewees, we modified some descriptions and wording in the questionnaire to make it easier to understand while maintaining the original meaning.

Before surveying, we submitted the application to the university and received approval from the academic board. We then distributed the modified questionnaires to older people in some residential communities in Northeast China. We worked with neighborhood committees who assisted us in recruiting participants, instructing participants to fill in the questionnaire, and collecting the responses. Before joining the study, all participants were informed the purpose of the survey and voluntarily choose whether to participate. To ensure the accuracy and validity, interviewees in the polit test were excluded. Once they agreed to participate, participants were given a paper questionnaire, along with a research staff who explains precautions and assists in filling it. The survey was anonymous, and participants were assured that the data collected will be kept confidential and used only for academic research, which encourages participants to answer the questionnaire as truthfully as possible. The survey was conducted on-site, where participants were rewarded with two eggs after completing the questionnaire. Completed questionnaires were collected and sent back directly to our research team for quality review and data analysis.

Out of 500 questionnaires that were distributed, 405 questionnaires were obtained after removing incomplete responses, rendering an 81% response rate. Since the objective of this study concerned the older segment of the population, respondents who were younger than 55 years old were excluded. The rationale is that the legal retirement age for females in China is 55, which is also widely identified as the age of older adults in numerous studies (63–65). Additionally, the questionnaire began with the screening question, “Have you experience in seeking health information using information technology or on the Internet?” If a respondent answered, “No,” the questionnaire was considered invalid. To further improve the validity of the questionnaires, we eliminated questionnaires that repeated more than 75 percent of the answers. A final total of 347 valid questionnaires was obtained for further analysis. We compared the demographics such as age, gender, and education between first 100 and last 100 respondents and found no significant differences, indicating that non-response bias was not a factor in this study. Table 1 presents the demographics information of the respondents.

**Table 1:** Demographic statistics.

**Variables**	**Category**	**Frequency**	**Percentage (%)**
Gender	Male	126	36.3
	Female	221	63.7
Age	55–60	40	11.5
	61–65	78	22.5
	66–70	89	25.6
	70–75	100	28.8
	76–80	36	10.4
	Over 80	4	1.2
Education level	Middle school and below	41	11.8
	High school	185	53.5
	College	85	24.5
	Bachelor's degree and above	36	10.4
Chronic disease	Yes	214	61.7
	No	133	38.3

### 4.2. Measurement

All measures of constructs in this study were adapted from previous studies and were appropriately modified to fit the current research context. Specifically, online health information seeking (OHIS) was measured with three items adapted from Cao et al. (66). Perceived benefit (PB) refers to older adults' perceived benefit of using IT to seek health information, which was measured with three items adapted from Al-Debei et al. (47). In addition, health awareness (HA) was measured with items adapted from Guo et al. (67), reflecting the health concerns and consciousness of the older adults. IT self-efficacy (ITS) and IT innovativeness (ITI), two constructs representing the personal IT resources of the study participants, were measured with items adapted from Thatcher and Perrewe (68) and Zhang et al. (69), respectively. Social support (SS) refers to the support that the participants received from family, friends, and social networks, which was measured with items adopted from Zimet et al. (70), while professional support (PS) refers to support from doctors and other medical professionals and was measured with items from Rosland et al. (71). All measurement items are specifically listed in the Table A1. Seven-point Likert scales were employed, ranging from 1 (strongly disagree) to 7 (strongly agree).

## 5. Data analysis and results

In this study, we used structural equation modeling (SEM) with the partial least squares (PLS) algorithm to analyze the collected data and evaluate the research model. PLS-SEM is relatively robust in survey data analysis while considered more suitable for testing models with small sample sizes (72); therefore, this method was deemed suitable for this study. SmartPLS 3.2 software was employed as our analytic tool. Following two-step procedures, we first examined the measurement model to ensure its reliability and validity, then examined the structural model to confirm the hypothesized relationships.

### 5.1. Measurement model

To assess the measurement model, we examined the reliability, convergent validity, and discriminant validity of our constructs. The reliability of constructs was assessed by checking whether composite reliability and Cronbach's alpha were higher than the threshold of 0.7. As shown in Table 2, composite reliability and Cronbach's alpha of all constructs were >0.7, indicating good reliability (73). Convergent validity was assessed by the item loadings and the average variance extracted (AVE) from expected constructs, which needed to be higher than 0.7 and 0.5, respectively (73). Table 3 reveals that all item loadings of constructs were >0.7; meanwhile, Table 2 shows that all AVE values were >0.5, thereby suggesting good convergent validity. Two approaches were employed to assess the discriminant validity. First, we compared whether the square root of AVE for a construct was greater than the correlation coefficients between the expected construct and other constructs. As shown in Table 2, all constructs satisfied the criterion. Second, we compared whether the item loadings of a construct were higher than the cross-loadings, which was verified by the results presented in Table 3. These results indicated that constructs in this study had good discriminant validities (74, 75).

**Table 2:** Reliabilities and correlations.

	**Cronbach's alpha**	**Composite reliability**	**Average variance extracted (AVE)**	**ITI**	**ITS**	**HA**	**OHIS**	**PB**	**PS**	**SS**
ITI	0.942	0.963	0.896	**0.947**						
ITS	0.92	0.949	0.862	0.722	**0.929**					
HA	0.902	0.939	0.837	0.284	0.221	**0.915**				
OHIS	0.879	0.926	0.806	0.635	0.667	0.299	**0.898**			
PB	0.893	0.934	0.825	0.714	0.652	0.236	0.611	**0.908**		
PS	0.834	0.901	0.753	0.5	0.471	0.341	0.636	0.5	**0.868**	
SS	0.901	0.938	0.834	0.471	0.339	0.428	0.39	0.445	0.274	**0.913**

Bold values refer to the square roots of AVE.

**Table 3:** Loadings and cross-loadings.

	**HA**	**ITI**	**ITS**	**OHIS**	**PB**	**PS**	**SS**
HA1	**0.883**	0.25	0.23	0.288	0.241	0.37	0.352
HA2	**0.939**	0.256	0.199	0.267	0.2	0.289	0.408
HA3	**0.921**	0.272	0.178	0.267	0.207	0.275	0.414
ITI1	0.26	**0.934**	0.706	0.616	0.67	0.507	0.447
ITI2	0.268	**0.942**	0.647	0.558	0.684	0.414	0.437
ITI3	0.277	**0.964**	0.699	0.638	0.674	0.5	0.453
ITS1	0.198	0.653	**0.94**	0.58	0.595	0.398	0.321
ITS2	0.186	0.65	**0.932**	0.635	0.593	0.467	0.277
ITS3	0.231	0.706	**0.913**	0.644	0.627	0.448	0.345
OHIS1	0.204	0.435	0.524	**0.817**	0.431	0.595	0.3
OHIS2	0.274	0.601	0.63	**0.934**	0.599	0.672	0.387
OHIS3	0.316	0.653	0.634	**0.935**	0.599	0.709	0.358
PB1	0.261	0.611	0.482	0.445	**0.846**	0.4	0.449
PB2	0.216	0.649	0.637	0.598	**0.932**	0.47	0.368
PB3	0.179	0.684	0.642	0.613	**0.943**	0.486	0.407
PS1	0.286	0.489	0.512	0.673	0.461	**0.896**	0.244
PS2	0.311	0.421	0.409	0.654	0.392	**0.904**	0.221
PS3	0.288	0.392	0.306	0.487	0.451	**0.798**	0.249
SS1	0.299	0.362	0.312	0.331	0.38	0.262	**0.852**
SS2	0.428	0.451	0.314	0.374	0.418	0.256	**0.94**
SS3	0.424	0.464	0.308	0.364	0.419	0.24	**0.944**

Bold values refer to item loadings of the corresponding construct.

To further test the potential problem of multi-collinearity for constructs, we calculated variance inflation factor (VIF) values. According to the results, VIF values for all constructs ranged from 1.059 to 2.052, less than the suggested criteria threshold of 3.3 (76). Thus, multi-collinearity was not an issue in this study. In addition, to test for common method bias, we used Harman's single factor test to examine whether a single component accounted for most of the variance (77). The results indicated that the most variance explained by one factor was 36.8%, which was lower than the 50% threshold, thus indicating that common method bias is not a concern.

### 5.2. Structural model

**Figure 2:**
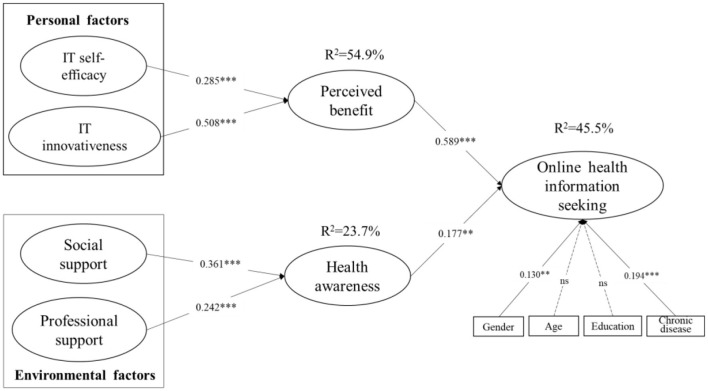
Structural model results. ***p < 0.001, **p < 0.01.

Figure 2 depicts the structural model results. For personal factors, both IT self-efficacy (β = 0.285, t = 4.621, *p* < 0.001) and IT innovativeness (β = 0.508, t = 9.168, *p* < 0.001) had a positive significant effect on perceived benefit, supporting H1 and H2. The results demonstrate that perceived benefit (β = 0.589, t = 13.373, *p* < 0.001) significantly promoted online health information seeking behavior, supporting H3. In addition, for environmental factors, social support (β = 0.361, t = 6.811, *p* < 0.001) and professional support (β = 0.242, t = 4.284, *p* < 0.001) were verified to positively impact health awareness, supporting H4 and H5, respectively. Meanwhile, health awareness (β = 0.177, t = 3.726, *p* < 0.001) showed a positive effect on online health information seeking. Overall, the structural model explained 45.5% of the variance in online health information seeking, along with 54.9% of the variance in perceived benefit and 23.7% of the variance in health awareness. Lastly, among the control variables, gender and chronic disease revealed a positive effect on online information seeking, indicating that female older adults and older adults with chronic disease were more likely to seek health information online.

### 5.3. *Post-hoc* analysis

The structural model results indicated that perceived benefit and health awareness simultaneously determined online health information seeking; specifically, perceived benefit was influenced by IT self-efficacy and IT innovativeness, while health awareness was influenced by social support and professional support. To further reveal the underlying influence mechanism, we went on to examine whether mediating effects existed in the research model. Employing the PROCESS, a widely used tool developed by Hayes (78) to estimate models with mediators, we tested the mediation effect of perceived benefit and health awareness. As presented in Table 4, both perceived benefit and health awareness demonstrated significant partial mediating effects. Specifically, the effects of IT self-efficacy and IT innovativeness on online health information seeking were partially mediated by perceived benefit. Similarly, the effects of social support and professional support on online health information seeking were partially mediated by health awareness.

**Table 4:** Mediation effect test.

**Mediator**	**Path**	**Indirect effect (95%)**			**Direct effect (95%)**			**Results**
		**Size**	**LLCI**	**ULCI**	**Size**	**LLCI**	**ULCI**	
PB	ITS->PB->OHIS	0.186	0.1090	0.2625	0.463	0.3558	0.5592	Patrial mediating
	ITI->PB->OHIS	0.215	0.1189	0.3069	0.399	0.2887	0.5090	Partial mediating
HA	SS->HA->OHIS	0.067	0.0201	0.1144	0.315	0.2107	0.4195	Partial mediating
	PS->HA->OHIS	0.038	0.0067	0.0481	0.474	0.4933	0.6564	Partial mediating

Furthermore, the results for the structural model visually demonstrated that the influence path coefficient (β = 0.589) between perceived benefit and online health information seeking was greater than the path coefficient (β = 0.177) between health awareness and online health information seeking. We further statistically verified whether there were differences in the effects of perceived benefit and health awareness on online health information seeking. Using the approach proposed by Keil et al. (79), we discovered that the difference in path coefficients between perceived benefit and health awareness on online health information seeking was significant (t = 116.384). Thus, perceived benefit was shown to play a more important role than health awareness in promoting older adults to seek health information online. Similarly, we also compared the effects of IT self-efficacy and IT innovativeness on perceived benefit, as well as the effects of social support and professional support on health awareness. The results in Table 5 indicate that IT innovativeness exerted a stronger impact than IT self-efficacy, while social support exhibited a stronger impact than professional support.

**Table 5:** Path coefficients comparison.

**DV**	**Path**	**Path coefficient**	**T value**	**Conclusion**
OHIS	β_PB−>OHIS_ vs. β_HA−>OHIS_	0.589^***^ vs. 0.177^***^	116.384^***^	β_PB−>OHIS_ > β_HA−>OHIS_
PB	β_ITS−>PB_ vs. β_ITI−>PB_	0.285^***^ vs. 0.508^***^	50.135^***^	β_ITS−>PB_ < β_ITI−>PB_
HA	β_SS>HA_ vs. β_PS−>HA_	0.361^***^ vs. 0.242^***^	4.755^***^	β_SS>HA_ > β_PS−>HA_

^***^p < 0.001.

## 6. Discussion

### 6.1. Key findings

Drawing upon social cognitive theory, this study investigated the effects of personal and environmental factors on the online behavior of older adults seeking health information and uncovered the influencing mechanism. The results elicit several key findings. For example, the study findings verified that IT self-efficacy and IT innovativeness are two crucial personal factors for older adults in promoting their online behavior when seeking health information; in particular, IT innovativeness was identified as having a stronger impact. This finding is in line with practice and previous studies that have emphasized the importance of IT capacity and resources in older adults' behavior related to searching for health-related information online (8).

This study also confirmed two significant environmental factors: social support and professional support. Our results indicated that both factors showed positive impacts whereby the effect of social support was stronger, demonstrating that support from doctors, families, and friends can encourage older adults to actively seek health information. This observation complements the findings of previous studies that less communication with professionals and families leads to online health information seeking (21, 80). We empirically found that support from professionals and families was significantly positively related to older adults' online health information seeking.

Our results also validate the direct effect and mediating role of perceived benefit and health awareness for older adults. Specifically, when such individuals believe that using IT is beneficial and experience a high level of health awareness, they tend to seek health information online. In particular, the perceived benefit of using IT to seek health information revealed a much stronger impact on older adults' behavior than health awareness. Furthermore, the effects of IT self-efficacy and IT innovativeness on online health information seeking were partially mediated by perceived benefit, while the effects of social support and professional support on online health information seeking were partially mediated by health awareness.

### 6.2. Theoretical implications

This study contributes to the field by raising several theoretical implications. First, this study enriches the research on online health information seeking through investigating older adults' online health information seeking behavior. Although a few scholars previously sought to understand older adults' attitudes toward searching for health information online (80, 81), they mainly explored and summarized the factors influencing older adults' behavior while neglecting to interpret how these factors motivated their subjects to seek health information online. As far as we know, our study is one of the first to address this issue. In particular, due to older adults' characteristics, their means of obtaining health information is usually passive when compared to young people (82). Therefore, clarifying the mechanism underlying older adults' online health information seeking can shed light on how to effectively facilitate this process for them while, at the same time, deepening the scholarly understanding of this issue.

Second, this study empirically confirms the antecedents of online health information seeking by contextualizing older adults' specific drivers. Drawing on social cognitive theory, we integrally examined antecedents from personal and environmental perspectives. Although factors such as IT self-efficacy and social support have been identified as playing significant roles in determining online health information seeking (10, 66), this study takes a further step by empirically verifying their effects on older adults' behavior. Based on the framework of social cognitive theory, we also authenticate the significant role of IT innovativeness and professional support. Furthermore, we clarify the differential impacts of antecedents by comparing their effects, revealing the underlying influence paths. Thus, this study not only comprehensively highlights the impacts of different determinants but also provides new understanding and suggests directions for future research.

Third, this study contributes to social cognitive theory by introducing it in the online health information seeking context and validating the mediation role of perceived benefit and health awareness. Although social cognitive theory has been widely applied in studies examining health behaviors (83, 84), to our knowledge, no other scholars have previously investigated online health information seeking from the perspective of social cognitive theory. This study fills a gap in the literature by providing a deeper understanding of how older adults' personal factors and environmental factors comprehensively influence how they search for health information online. Furthermore, this study certifies that older adults' cognitions and perceptions (i.e., perceived benefit and health awareness) significantly mediate the impacts of personal factors and environmental factors on behavior. In this regard, this study enriches the previous understanding of social cognitive theory by revealing the influence mechanism of personal and environmental factors.

### 6.3. Practical implications

The findings of this study lead to some practical suggestions to aid the public, especially older adults, in actively seeking health information online. For example, results illustrated that both perceived benefit and health awareness significantly enhance older adults' search behavior, suggesting managers and organizations should take corresponding measures to improve older adults' evaluation of information technology and promote their health awareness. Accordingly, to enhance perceived benefit, health information technology service providers are encouraged to develop and optimize health information seeking functions to improve user-friendliness, such as increasing front size, simplifying search interface, and adding guidance-related notes for older adults. These tips will improve user-friendliness and are likely to increase older adults' perceived benefit and further promote online health information seeking. In addition, since health awareness is positively related to health information seeking behavior, organizations and governments are encouraged to organize health lectures and strengthen health publicity to improve older adults' health awareness.

Moreover, IT self-efficacy has been found to play a significant role in older adults' perceived benefit and online health information seeking, thus practitioners should address the issue of improving older adults' IT-related abilities. Along these lines, health information technology companies are advised to set up a special department for older adult users and send staff to train such individuals on how to effectively use the Internet to obtain health information. Furthermore, since IT innovativeness significantly increases perceived benefit and further promotes information seeking, we recommend that information technology designers should develop exploratory features for older adults to improve their innovativeness. For example, developing exploratory games and displaying them on login screens to encourage older adults act in a more innovative way.

Lastly, results in this study demonstrated that social support and professional support significantly enhance older adults' health awareness and further facilitate their efforts to find relevant health information. Therefore, we strongly suggest that healthcare workers and people surrounding older adults (e.g., family and friends) should provide more support in terms of their health management. According to our findings, a family doctor is necessary for older adults, allowing them to obtain professional information and medical support on a regular basis. Similarly, since social support was shown to have a stronger positive impact on older adults' health awareness, we suggest that families and friends offer more help and care to older adults, for example, keeping an eye on their health and discussing health issues with them regularly.

### 6.4. Limitations and future research

Although several notable theoretical and practical implications emerged from the study findings, some limitations should be addressed, which lead to suggestions for future research. For example, this study used a cross-sectional survey to examine the research model. Although tests were conducted to verify that the results were not affected by common method bias and multicollinearity, future research is strongly recommended using mixed methods and a longitudinal design to confirm the causal relationships. Furthermore, the research was conducted on the Chinese mainland, potentially limiting the generality of the research results. In particular, older Chinese adults prefer collectivism in which social support is more likely to play significant roles. Future research should therefore take cross-cultural issues into account to reach more interesting findings. Lastly, drawing on social cognitive theory, we captured IT self-efficacy and IT innovativeness as personal factors and incorporated professional support and social support as environmental factors. However, technology characteristics, such as the user-friendliness of information technology, may also play significant roles and are worth investigating in future research.

## Data availability statement

The original contributions presented in the study are included in the article/supplementary material, further inquiries can be directed to the corresponding author.

## Ethics statement

The studies involving human participants were reviewed and approved by Ethical Committee of the Harbin Institute of Technology (N.2021-10 dated on 8th of November 2021). The patients/participants provided their written informed consent to participate in this study.

## Author contributions

XM and PZ: conceptualization, methodology, and writing. YL and RQ: methodology and writing. FM: review, editing, and supervision. All authors contributed to the article and approved the submitted version.

## Footnotes

^1^ https://www.who.int/news-room/fact-sheets/detail/ageing-and-health

## Appendix

**TABLE A1:** Survey instruments.

**Construct**	**Questions**	**Reference**
Online health information seeking	I frequently find health information online.	(66)
	I often use health websites.	
	I often seek health information on the internet.	
Perceived benefit	By using internet, I can seek health information in privacy conveniently.	(47)
	I can seek health information online whenever I want.	
	Seeking health information online can save me the time and effort.	
Health awareness	I reflect about my health a lot.	(67)
	I am very self-conscious about my health.	
	I am generally attentive to my inner feelings about my health.	
IT self-efficacy	I can complete the task such as seeking health information using internet if… (1) …there was no one around to tell me what to do	(68)
	(2) …I had seen someone else using it before trying it myself.	
	(3) …someone showed me how to do it first.	
IT innovativeness	I am willing to try new information technologies.	(69)
	I think it is very interesting to try new information technologies.	
	I enjoy trying new information technologies.	
Social support	I can talk about my health problems with my family and friends.	(70)
	My family and friends are willing to help me in my health.	
	I get the help and support I need from my family and friends.	
Professional support	I can get the support I need from doctors and other health professionals.	(71)
	I can account on doctors to help and support me a lot with health problems.	
	I am satisfied with support from doctors and other health professionals.	
